# Impact of Mutational Status and Prognostic Factors on Survival in Chronic Myelomonocytic Leukemia With Systemic Inflammation and Autoimmune Disorders

**DOI:** 10.1097/HS9.0000000000000847

**Published:** 2023-02-23

**Authors:** Charles Dussiau, Henry Dupuy, Audrey Bidet, Mathieu Sauvezie, Anne-Charlotte De-Grande, Lisa Boureau, Etienne Riviere, Edouard Forcade, Fabrice Bonnet, Pierre-Yves Dumas, Pierre Duffau, Arnaud Pigneux, Jean-François Viallard, Sophie Dimicoli-Salazar, Estibaliz Lazaro

**Affiliations:** 1 Department of Hematology Biology, Molecular Hematology, Bordeaux University Hospital, Haut-Levêque Hospital, Pessac, France; 2 Department of Internal Medicine and Infectious Diseases, Bordeaux University Hospital, Haut-Levêque Hospital, Pessac, France; 3 Department of Clinical Hematology and Cell Therapy, Bordeaux University Hospital, Haut-Haut-Levêque Hospital, Pessac, France; 4 Department of Internal Medicine and Infectious Diseases, Bordeaux University Hospital, Saint André Hospital, Bordeaux, France; 5 Department of Internal Medicine and Clinical Immunology, Bordeaux University Hospital, Saint André Hospital, Bordeaux, France

Chronic myelomonocytic leukemia (CMML) is a clonal hematopoietic malignancy with features of both myeloproliferative neoplasms and myelodysplastic neoplasms (MDS). MDS are associated in 10%–20% of cases with various systemic inflammation and autoimmune disorders (SIAD).^1^ Like MDS, CMML is associated with SIAD in approximately 30% of cases.^2^ Previously, we have shown that inflammatory pathways responsible for SIAD in CMML were not linked with the *UBA1* mutation and could depend on its mutational profile.^3^ Studies regarding mutational status and survival that focus solely on CMML with SIAD manifestations are scarce and do not accurately differentiate between SIAD subtypes.^2,4,5^

We report here a large French retrospective single-center cohort of CMML patients referred to our academic center between January 1999 and December 2019, from which we analyzed the clinical and biological characteristics, mutational profile, prognostic factors, and survival according to the different SIAD subtypes. The diagnosis of CMML was retained according to the 2016 WHO classification.^6^ Molecular analyses were performed on bone marrow samples using a next-generation sequencing panel of 64 genes involved in myeloid malignancies (Suppl. Table S1). SIAD was defined according to international criteria and classified as previously described^7^ (Suppl. data: Methods).

A total of 131 CMML patients were included in this cohort, and 47 had SIAD (36%). Regarding baseline clinical and biological characteristics, no significant differences were found between patients with SIAD and those without SIAD (Suppl. Table S2). Since several different subtypes of SIAD can be present in the same patient, we observed a total of 71 SIAD (Suppl. Table S3).

The main SIAD manifestations that account for 75 % of them all were 24 autoimmune cytopenias (34%), 14 inflammatory skin diseases (19%), 9 inflammatory arthritis (13%), and 6 pericardial effusions (9%). Among the 24 autoimmune cytopenias, there were 18 immune thrombocytopenia (ITP) (75%). Excluding 1 pediatric ITP, the median time from ITP diagnosis to CMML diagnosis was 24 months (range 244–0). The median platelet count at ITP diagnosis was 14 × 10^9^/L. On the 17 bone marrow analyses available, megakaryocyte count was normal or increased in 88% of the cases, and no significant dysmegakaryopoiesis was retained. On the 11 treated ITP, 63% were responsive to corticosteroid therapy and/or intravenous immunoglobulin.

Regarding skin lesions, there were 4 neutrophilic dermatoses (29%), 6 specific inflammatory skin lesions including annular erythema, pemphigus vulgaris, cutaneous vasculitis, chronic pruritus and psoriasis, and 4 aspecific skin lesions (profuse keratosis, ulcerative necrosis, and papulonodular lesions) without histologically proven localizations of CMML. Skin lesions appeared concomitantly with CMML diagnosis (median delay of 0 month, range 72 months before CMML diagnosis to 12 months after). All patients with CMML and polyarthritis had large and peripheral joints affected. The polyarthritis (except 2 patients with prior rheumatoid arthritis) occurred simultaneously to CMML. Five patients with pericarditis had emergency drainage of an inflammatory pericardial fluid. The diagnostic work-up of pericarditis was negative for connective tissue disease, infectious or neoplastic disease and lead to CMML diagnosis. Four cases of pericarditis occurred simultaneously with acute increase in monocytes (up to 20 G/L). None of these previously described patients received immunosuppressive therapy responsible for myelotoxicity, except the 2 rheumatoid arthritis treated by methotrexate. On the 18 other SIAD, the most relevant were 4 vasculitis (22%; giant cell arteritis, polyarteritis nodosa [PAN], multiple venous thrombosis, and multiple arterial thrombosis).

Autoimmune cytopenia was an isolated manifestation in more than 60% of cases and was rarely associated with other SIAD, while patients with cutaneous, rheumatologic, and cardiac manifestations often had several associated SIAD (Suppl. Figure S1).

Each SIAD subtype was compared to other SIADs. No significant differences were found except for platelet count, which was lower in the autoimmune cytopenia group than in the other patients with SIAD (70 versus 100 × 10^9^/L, *P* < 0.05; Suppl. Tables S4–S7).

Targeted sequencing revealed pathogenic mutations in 33 different genes. The mutational landscape of the 131 CMMLs showed a predominance of the following mutations: *TET2*^mut^ (73%)*, SRSF2*^*mut*^ (47%), and *ASXL1*^*mut*^ (39%). Other frequently mutated genes were *RUNX1*^*mut*^ (25%), *NRAS*^*mut*^ (20%), *KRAS*^*mut*^ (18%), *CBL*^*mut*^ (18%), and *DNMT3A*^*mut*^ (11%). No *UBA1* mutation was found (Suppl. Figure S2). No significant differences were found in the mutational landscape of patients with SIAD compared with non-SIAD patients (Suppl. Table S8). No genes were found to be significantly associated to a specific SIAD subtype (Suppl. Tabl S9–S12).

In the whole cohort, the median overall survival (OS) was 3.2 years (95% confidence interval, 2.25-4.76 years). As expected, OS and progression-free survival (PFS) in the entire cohort were significantly different according to CPSS (CMML-Specific Prognostic Scoring System) and CPSS-Mol (CPSS molecular) scores, with a poorer prognosis in patients with a high CPSS and CPSS-Mol score (Suppl. Figure S3). According to a univariate Cox model, the presence of SIAD had no prognostic impact on survival. In the multivariate Cox model, factors associated with a better OS were MD-CMML and *TET2*^*mut*^ (*P* < 0.005). A better PFS was also observed in MD-CMML patients (*P* = 0.0052), whereas *RUNX1*^*mut*^ was associated with a poorer PFS (*P* < 0.005; Suppl. Tables S13, S14).

Regarding only SIAD patients, no significant differences were found in OS and PFS according to CPSS and CPSS-Mol scores (Figure 1). In multivariate Cox analysis, autoimmune cytopenia (20 patients with AI cytopenia, 27 without) was associated with a better OS contrary to *DNMT3A*^*mut*^ (4 *DNMT3A*^*mut*^ patients and 43 *DNMT3A*^*wt*^). Factors significantly associated with worse PFS were *ASXL1*^*mut*^, *CUX1*^*mut*^, and *DNMT3A*^*mut*^ (Figure 2, Suppl. Tables 15, 16).

**Figure 1.**
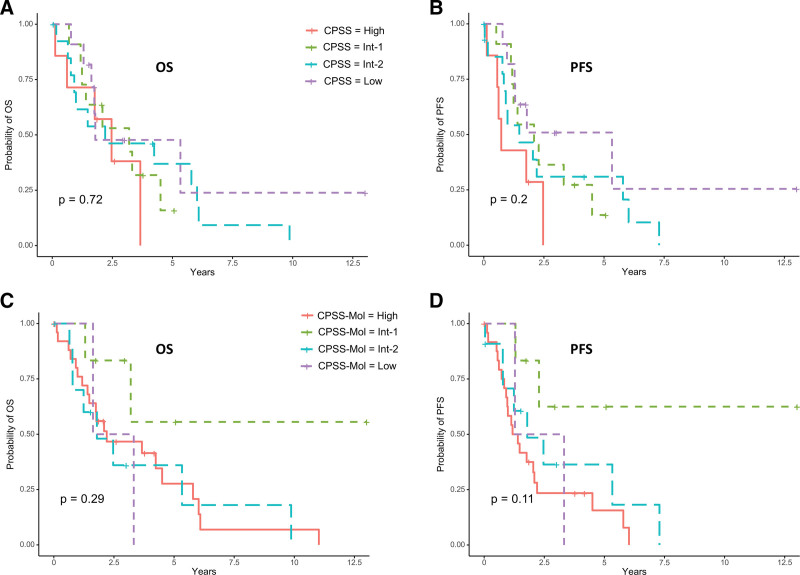
**Survival analysis of CMML patients with SIAD according to CPSS and CPSS-mol risk score.** (A) OS and (B) PFS of patients with SIAD according to the CPSS risk score. Distribution of patients in the different groups of the CPSS risk score: 7 high, 15 intermediate-2, 13 intermediate-1, and 11 low. (C) OS and (D) PFS of patients with SIAD according to the CPSS-Mol risk score. Distribution of patients in the different groups of the CPSS-Mol risk score: 26 high, 11 intermediate-2, 6 intermediate-1, and 2 low. Survival curves were compared with the log-rank test. CPSS = CMML-Specific Prognostic Scoring System; OS = overall survival; PFS = progression-free survival; SIAD = systemic inflammation and autoimmune disorders.

**Figure 2.**
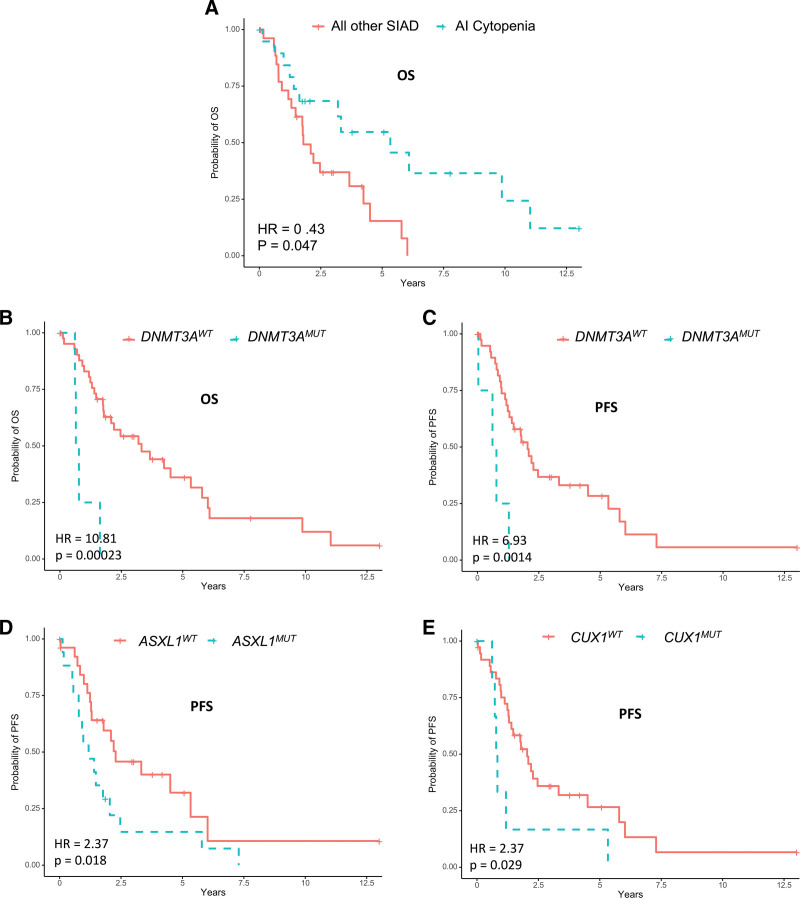
**Significant prognostic factors for survival in CMML patients with SIAD.** (A) OS of SIAD patients according to the autoimmune cytopenia status (20 patients with AI cytopenia, 27 without). (B) OS and (C) PFS of SIAD patients according to *DNMT3A* mutational status (4 *DNMT3A*^*mut*^ patients and 43 *DNMT3A*^*wt*^). (D) PFS of SIAD patients according to *ASXL1* mutational status (17 *ASXL1*^*mut*^ patients and 30 *ASXL1*^*wt*^). (E) PFS of SIAD patients according to CUX1 mutational status (7 *CUX1*^*mut*^ patients and 40 *CUX1*^*wt*^). For each panel, HR, and *P* value were computed with the multivariate Cox regression model. HR = hazard ratio; OS = overall survival; PFS = progression-free survival; SIAD = systemic inflammation and autoimmune disorders.

Our study confirms the diversity and high frequency of SIAD associated with CMML, reaching 36% in our retrospective cohort. The most prevalent were autoimmune cytopenias (34%), inflammatory skin diseases (19%), inflammatory arthritis (13%), pericardial effusions (9%), and few cases of systemic vasculitis (6%). Previous studies reported a prevalence of ITP in 29% of CMML with SIAD, whereas inflammatory skin diseases, mostly neutrophilic dermatoses, and inflammatory arthritis were reported up to 15%.^8–10^ Cases of pericarditis associated with CMML without pericardial monocyte infiltration have been described.^11^ Some cohorts reported a prevalence of vasculitis in up to half of CMML patients with SIAD, mostly PAN.^10,12^ Discerning causality or coincidence regarding the association of SIAD and CMML is difficult, notably when SIAD precedes CMML by several years. In our cohort, rheumatologic, skin, and heart SIAD were diagnosed simultaneously with CMML for the majority of cases, probably because these manifestations lead to a broad diagnostic approach.

Regarding prognosis, in the whole cohort, SIAD were not associated with a pejorative prognosis, in accordance with other studies.^10,13^ We showed that among CMML patients with SIAD, autoimmune cytopenia had a significantly better OS compared with other types of SIAD. The similar mutational landscape between each SIAD subtype suggests that this difference may not be explained by mutations studied in our work.

In our study, *DNMT3A* mutation is a predictor of poor survival in terms of OS and PFS only in patients with SIAD, but not in the entire cohort, contrary to what was reported in a dedicated study.^14^ These data suggest that the *DNMTA3* status merits to be analyzed in larger cohorts of CMML patients with SIAD as it seems to impact their prognosis.

CPSS and CPSS-Mol scores, considered as the most relevant scoring systems for CMML in a contemporary practice, were not applicable in SIAD patients.^13^ This result suggests that even though the mutational landscape was similar in CMML patients with or without SIAD, other factors have an impact on these scores.

In conclusion, this study reveals the high frequency of SIAD associated with CMML and the specific prognostic factors of these patients. CMML with SIAD have a better prognosis in case of autoimmune cytopenia and *DNMT3A, ASXL1*, and *CUX1* mutations are pejorative mutations in terms of prognosis. It could be interesting to compare survival of SIAD patients with CMML to SIAD patients without CMML, and to look for clonal hematopoiesis in these SIAD patients. CPSS and CPSS-Mol are not applicable to CMML patients with SIAD, indicating the need for other prognostic scoring systems for these patients.

## ACKNOWLEDGMENTS

We thank Dr. David A. Lawrence for assistance with the manuscript. This work was realized thanks to the sequencing facility sponsored by the Nouvelle Aquitaine Region (grant number 2018-1R30113-8473520).Author ContributionsCD, HD, SDS, EL designed the work, performed the data collection, analysis and interpretation, drafted the article and approved the final version to be published. AB, MS, ACDG, LB, ER, EF, FB, PYD, PD, AP and JFV provided critical feedback.

## DISCLOSURES

The authors have no conflicts of interest to disclose.

## Supplementary Material


