# Supplementary material for: Impact of Mutational Status and Prognostic Factors on Survival in Chronic Myelomonocytic Leukemia With Systemic Inflammation and Autoimmune Disorders
Source: Hemasphere. 2023 Feb 23;7(3):e847. doi: 10.1097/HS9.0000000000000847 (PMC9953038; doi:10.1097/HS9.0000000000000847)
Supplement: Supplementary file 1 [file hs9-7-e847-s001.docx]

**Supplementary data**

**Methods:**

**Patients:**

We conducted a retrospective single-center cohort study at the Bordeaux University Hospital in France. Diagnosis of CMML was retained according to the 2016 WHO classification, namely a chronic non-reactive monocytosis, an absence of myeloproliferative neoplasm, a blood and marrow blast cell count of less than 20% and a dysplasia of one of the myeloid lineages on the bone marrow aspiration. CMMLs were classified according to their stage 0, 1 or 2, to their myelodysplastic (MD-CMML) or myeloproliferative (MP-CMML) profile, and to the CPSS (CMML-specific prognostic scoring system) and CPSS-Mol prognostic scoring systems.

Data were obtained from patients in the DATAMDS Bordeaux Registry which includes 500 patients with MDS and CMML newly diagnosed between January 1, 1999, and December 2019. This study was conducted in accordance with the French law MR-004 delimiting research on medical files and was approved by the local ethics committee (CNIL number: VEc2491306D)

**Clinical and laboratory assessments**

The following data collected at the time of CMML diagnosis were included: age, sex, CMML type according to the WHO classification, and other laboratory data including white blood cell, platelet counts and hemoglobin.

Molecular analyses were performed using DNA extracted from archived frozen cytogenetic cell pellets or total leukocytes from bone marrow using Maxwell RSC Instrument (Promega).

. Detection of somatic mutations was performed by the Laboratory of Hematology of the University Hospital of Bordeaux using a next-generation sequencing (NGS) panel designed for myeloid hematological malignancies. Eight other genes already described in auto-immune cytopenias have been added into the panel: *TNFRSF6B, CTLA4, UBA1, STAT3, PIK3CD, ADAR, LRBA, RAG1*. The 64 genes tested are shown in Supplementary Table 1. Target sequences were captured using SureSelect technology. Sequencing was performed on a NextSeq 550Dx Instrument and analyzed using an in-house bioinformatics pipeline:

*Library preparation and sequencing*

A custom RNA-baits panel was designed to cover 64 genes involved in myeloid malignancies. The list of the genomic regions targeted is available in the Supplementary Table 1. Libraries were prepared from approximatively 200 ng of DNA for each patient using SureSelect XT Low Input kit (Agilent) or using Magnis NGS Prep System (Agilent). Libraries were pooled for multiplex sequencing on a NextSeq500 (Illumina) with Mid Output Kit v2.

*Bioinformatic pipeline*

We developed a bioinformatic pipeline to analyse sequencing data in order to control each step of analysis. FASTQ files were generated by bcl2fastq. They were then aligned against reference genome hg19 (2013) with bwa-mem, producing BAM files. After this step, duplicate reads were tagged but not removed using agilent locatit software. Finally, coverage analysis was performed with bbctools, mosdepth, samtools and resulting metrics were gathered with MultiQC to assess sequencing data quality, including depth of coverage for every gene of panel. For this study we used 3 different tools for variant calling in order to detect with good accuracy mutations in samples. We have chosen GATK Mutect2, VarScan and VarDict to detect somatic mutations. Annovar software (version 2020-06-08) and ensembl VEP (v.103) were used to annotate all called variants. Following databases were used:

- COSMIC 92 (Catalogue Of Somatic Mutations In Cancer)

- gnomAD 2.1.1 (The Genome Aggregation Database), ExaC (The Exome Aggregation Consortium), 1000 genome, ESP (Exome Sequencing Project) to assess variant frequency in world population

- SIFT, PolyPhen2, PROVEAN (in-silico pathology prediction tools)

- dbSNP (The Single Nucleotide Polymorphism database)

- ClinVar (2021-01-03) (information on the relationships between variants and human health): this descriptor allows to know clinical effect of variant

- InterVar (software tool for automatic clinical interpretation of genetic variants by the ACMG/AMP 2015 guideline)

*Review and classification of mutations*

Intronic and synonymous mutations were removed as well as variants with VAF <1% and variants with a minor allele frequency (MAF) ≥0.1% listed in databases (dbSNP, gnomAD, ExaC, 1000 genome, ESP). Variants known as recurring artifact (manually curated and stocked in a local database) were also removed.

Finally, retained variant were reviewed independently by 2 molecular biologists for (i) visual inspection of reads in BAM file to conclude for real mutation or artifact and for (ii) classification of the pathogenicity of mutations. To be interpretable, the generated data had to meet the following criteria:

- Minimum read depth of 200X

- Sufficient coverage of the different regions in both reading directions

- Total number of reads corresponding to the variant must be greater than 10

In order to have more confidence in variants with low VAF, we distinguished them from artifacts by estimating the background noise *via* the median absolut deviation and the corresponding confidence intervals. To exclude variants that could represent artifacts, we only retained those that presented a VAF at least 2-fold higher than the upper limit of the confidence interval.

The classification of mutations was based on the consensus recommendation of the Association for Molecular Pathology and the American Society of Clinical Oncology.

**Clinical assessment of the SIAD**

The medical records were systemically reviewed, and inflammatory and autoimmune disorders were recorded as SIAD for the following organs at the time of SIAD diagnosis and during follow-up: constitutional symptoms and non-infectious fever, lung, kidney, nervous system, skin, joint, eye or heart involvement, venous or arterial thrombosis and peripheral cytopenia.

**Statistical analysis**

All statistical analysis were performed with R software (version 4.0.3). Differences in quantitative variables between groups were assessed using Wilcoxon rank sum test. For qualitative variables the Pearson's Chi-squared or Fisher's exact test were used.

Overall survival (OS) was the time from diagnosis of CMML to the date of death or last follow up. Progression free survival (PFS) was the time from diagnosis to transformation into high risk CMML, acute myeloid leukemia, death, or last follow up. OS and PFS curves were estimated using the Kaplan-Meier method, and differences in subgroups were assessed by the Log-Rank test.

To identify prognostic factors of Overall survival and Progression free survival, a univariate Cox regression model was first performed. Only frequent variables (present in > 5% of each group of patients) with a p-value < 0.1 in univariate Cox model were included for multivariate analysis. The best multivariate Cox model was then selected using the Akaike’s Information Critera method. The proportional-hazards assumption was tested on the basis of Schoenfeld residuals.

All p-values are two-sided and p-values <0.05 were considered statistically significant. Adjusted p-values were calculated to take into account multiple testing using the Benjamini-Hochberg procedure.

**Tables**

| **Genes** | | |
| --- | --- | --- |
| ACE2 | IDH2 | SAMD9 |
| ADAR | IKZF1 | SAMD9L |
| ANKRD26 | IL3 | SETBP1 |
| ASXL1 | IL6 | SF3B1 |
| BCOR | JAK2 | SH2B3 |
| BCORL1 | KDM6A | SMC1A |
| CALR | KIT | SMC3 |
| CASP3 | KRAS | SRSF2 |
| CBL | LRBA | SRY |
| CEBPA | MPL | STAG2 |
| CSF3R | MYD88 | STAT3 |
| CTLA4 | NEDD8 | TERC |
| CUX1 | NPM1 | TERT |
| DDX41 | NRAS | TET2 |
| DNMT3A | PHF6 | TNFRSF6B |
| ETNK1 | PIK3CD | TP53 |
| ETV6 | PPMID | U2AF1 |
| EZH2 | PTEN | UBA1 |
| FLT3 | PTPN11 | WT1 |
| GATA1 | RAD21 | ZRSR2 |
| GATA2 | RAG1 |  |
| IDH1 | RUNX1 |  |

**Supplementary Table 1:** list of the 64 genes tested

| **Participants** | | **All, N = 131** | **No SIAD, N = 84** | **SIAD, N = 47** | **p** |
| --- | --- | --- | --- | --- | --- |
| **Demographic characteristics** | Male sex - N (%) | 78 (60%) | 54 (64%) | 24 (51%) | 0.6 |
|  | Median age at diagnosis (range) - yrs | 74 (48, 95) | 74 (48, 95) | 73 (53, 90) | >0.9 |
| **Laboratory findings –**  **Median (IQR)** | Leukocytes - x 10^9^/L | 10 (7, 19) | 10 (6, 16) | 13 (9, 21) | 0.4 |
|  | Neutrophils - x 10^9^/L | 6 (3, 10) | 5 (2, 10) | 7 (4, 11) | 0.4 |
|  | Platelets - x 10^9^/L | 95 (61, 160) | 93 (62, 182) | 99 (53, 153) | >0.9 |
|  | Hemoglobin - g/dL | 11.70 (9.95, 13.20) | 11.65 (9.90, 13.20) | 11.80 (10.05, 13.05) | >0.9 |
|  | Monocytes - x 10^9^/L | 2.3 (1.5, 4.4) | 2.2 (1.4, 4.6) | 2.6 (1.7, 4.2) | >0.9 |
| **Karyotype - N (%)** | Normal | 89 (76%) | 57 (76%) | 32 (76%) | >0.9 |
|  | 1 other abnormality | 10 (8.5%) | 8 (11%) | 2 (4.8%) |  |
|  | del (5q) | 1 (0.9%) | 1 (1.3%) | 0 (0%) |  |
|  | 2 other abnormalities | 1 (0.9%) | 1 (1.3%) | 0 (0%) |  |
|  | 8 trisomy | 8 (6.8%) | 5 (6.7%) | 3 (7.1%) |  |
|  | del (20q) | 5 (4.3%) | 2 (2.7%) | 3 (7.1%) |  |
|  | Complex | 1 (0.9%) | 0 (0%) | 1 (2.4%) |  |
|  | Abnormality of chromosome 5 | 1 (0.9%) | 1 (1.3%) | 0 (0%) |  |
|  | Abnormality of chromosome 3 | 1 (0.9%) | 0 (0%) | 1 (2.4%) |  |
|  | Unknown | 14 | 9 | 5 |  |
| **IPSS score - N (%)** | 1 | 31 (29%) | 16 (24%) | 15 (38%) | >0.9 |
|  | 2 | 49 (46%) | 33 (49%) | 16 (40%) |  |
|  | 3 | 26 (24%) | 17 (25%) | 9 (22%) |  |
|  | 4 | 1 (0.9%) | 1 (1.5%) | 0 (0%) |  |
|  | Unknown | 24 | 17 | 7 |  |
| **R-IPSS score - N (%)** | 1 | 18 (17%) | 11 (17%) | 7 (18%) | >0.9 |
|  | 2 | 34 (32%) | 19 (29%) | 15 (38%) |  |
|  | 3 | 29 (28%) | 18 (27%) | 11 (28%) |  |
|  | 4 | 23 (22%) | 17 (26%) | 6 (15%) |  |
|  | 5 | 1 (1.0%) | 1 (1.5%) | 0 (0%) |  |
|  | Unknown | 26 | 18 | 8 |  |
| **MD vs MP - N (%)** | MD-CMML | 81 (62%) | 57 (68%) | 24 (51%) | 0,4 |
|  | MP-CMML | 50 (38%) | 27 (32%) | 23 (49%) |  |
| **CPSS score - N (%)** | High | 22 (17%) | 15 (18%) | 7 (15%) | >0.9 |
|  | Intermediate 1 | 37 (29%) | 24 (29%) | 13 (28%) |  |
|  | Intermediate 2 | 37 (29%) | 22 (27%) | 15 (33%) |  |
|  | Low | 32 (25%) | 21 (26%) | 11 (24%) |  |
|  | Unknown | 3 | 2 | 1 |  |
| **CPSS-Mol score - N (%)** | High | 62 (50%) | 36 (45%) | 26 (58%) | >0.9 |
|  | Intermediate 1 | 26 (21%) | 20 (25%) | 6 (13%) |  |
|  | Intermediate 2 | 27 (22%) | 16 (20%) | 11 (24%) |  |
|  | Low | 10 (8.0%) | 8 (10%) | 2 (4.4%) |  |
|  | Unknown | 6 | 4 | 2 |  |
| **CMML-stage - N (%)** | CMML-0 | 34 (26%) | 21 (25%) | 13 (28%) | >0.9 |
|  | CMML-1 | 55 (42%) | 35 (42%) | 20 (43%) |  |
|  | CMML-2 | 42 (32%) | 28 (33%) | 14 (30%) |  |
| **SIAD - N (%)** | Inflammatory arthritis | 9 (6.9%) |  | 9 (19%) |  |
|  | Inflammatory skin disease | 12 (9.2%) |  | 12 (26%) |  |
|  | Pericarditis | 6 (4.6%) |  | 6 (13%) |  |
|  | Autoimmune cytopenia | 20 (15%) |  | 20 (43%) |  |
|  | Other | 17 (13%) |  | 17 (36%) |  |
| **Median delay between SIAD and CMML diagnosis - N (months)** | Arthritis (Excluding RA) | 0 (-6 to 6) |  |  |  |
|  | Inflammatory skin disease | 0 (-72 to 12) |  |  |  |
|  | Pericarditis | 0 (-6 to 6) |  |  |  |
|  | ITP | -24 (-244 to 0) |  |  |  |

**Supplementary Table 2:** Baseline characteristics of the CMML cohort and depending on SIAD status.

| **SIAD** | | |
| --- | --- | --- |
| **Autoimmune Cytopenias** | Immune thrombocytopenia | 18 |
|  | Warm autoimmune hemolytic anemia | 3 |
|  | Autoimmune neutropenia | 2 |
|  | Autoimmune erythroblastopenia | 1 |
|  | **Total** | **24** |
| **Inflammatory skin disease** | Neutrophilic dermatoses | 4 |
|  | Various skin lesions | 4 |
|  | Pemphigus vulgaris | 1 |
|  | Cutaneous vasculitis | 1 |
|  | Chronic Pruritis | 1 |
|  | Multidrug-resistant psoriasis | 2 |
|  | Annular erythema | 1 |
|  | **Total** | **14** |
| **Inflammatory arthritis** | Inflammatory arthritis | 7 |
|  | Rheumatoid arthritis | 2 |
|  | **Total** | **9** |
| **Pericarditis** | Pericardial effusion | 6 |
|  | **Total** | **6** |
| **Vasculitis** | Giant cell arteritis | 1 |
|  | Polyarteritis nodosa | 1 |
|  | Unexplained multiple venous thrombosis | 1 |
|  | Unexplained mutliple arterial thrombosis | 1 |
| **Other SIAD** | Chronic kidney failure and tubulopathy | 3 |
|  | Sjogren's syndrome | 2 |
|  | Myelitis, myasthenia gravis, mutiples sclerosis | 3 |
|  | Hashimoto thyroiditis | 1 |
|  | Diffuse interstitial lung disease | 1 |
|  | Sclerosing cholangitis | 1 |
|  | Crohn's disease | 1 |
|  |  |  |

**Supplementary Table 3:** Characteristics of SIAD

| **Participants** | | **All other SIAD, N = 27** | **Autoimmune cytopenia, N = 20** | **p** |
| --- | --- | --- | --- | --- |
| **Demographic characteristics** | Male sex - N (%) | 15 (56%) | 9 (45%) | 0.8 |
|  | Median age at diagnosis  (range) - yrs | 77 (69, 81) | 73 (70, 78) | 0.8 |
| **Laboratory findings –**  **Median (IQR)** | Leukocytes - x 10^9^/L | 15 (9, 21) | 12 (9, 16) | 0.8 |
|  | Neutrophils - x 10^9^/L | 7 (5, 14) | 7 (4, 9) | 0.8 |
|  | Platelets - x 10^9^/L | 141 (82, 176) | 70 (43, 95) | 0.049 |
|  | Hemoglobin - g/dL | 11.40 (9.90, 12.45) | 12.55 (10.25, 13.33) | 0.7 |
|  | Monocytes - x 10^9^/L | 2.5 (1.8, 4.4) | 2.6 (1.5, 3.9) | 0.8 |
| **Karyotype - N (%)** | Normal | 18 (75%) | 14 (78%) | 0.8 |
|  | 1 other abnormality | 1 (4.2%) | 1 (5.6%) |  |
|  | 8 trisomy | 2 (8.3%) | 1 (5.6%) |  |
|  | del (20q) | 3 (12%) | 0 (0%) |  |
|  | Complex | 0 (0%) | 1 (5.6%) |  |
|  | Abnormality of  chromosome 3 | 0 (0%) | 1 (5.6%) |  |
|  | Unknown | 3 | 2 |  |
| **IPSS score - N (%)** | 1 | 7 (32%) | 8 (44%) | >0.9 |
|  | 2 | 10 (45%) | 6 (33%) |  |
|  | 3 | 5 (23%) | 4 (22%) |  |
|  | Unknown | 5 | 2 |  |
| **R-IPSS score - N (%)** | 1 | 4 (18%) | 3 (18%) | >0.9 |
|  | 2 | 9 (41%) | 6 (35%) |  |
|  | 3 | 6 (27%) | 5 (29%) |  |
|  | 4 | 3 (14%) | 3 (18%) |  |
|  | Unknown | 5 | 3 |  |
| **SIAD - N (%)** | Inflammatory arthritis | 8 (30%) | 1 (5.0%) | 0.6 |
|  | Inflammatory skin disease | 11 (41%) | 1 (5.0%) | 0.089 |
|  | Pericarditis | 6 (22%) | 0 (0%) | 0.4 |
|  | Other | 12 (44%) | 5 (25%) | 0.7 |
| **MD vs MP - N (%)** | MD-CMML | 12 (44%) | 12 (60%) | 0.8 |
|  | MP-CMML | 15 (56%) | 8 (40%) |  |
| **CPSS score - N (%)** | High | 4 (15%) | 3 (16%) | 0.8 |
|  | Intermediate 1 | 5 (19%) | 8 (42%) |  |
|  | Intermediate 2 | 11 (41%) | 4 (21%) |  |
|  | Low | 7 (26%) | 4 (21%) |  |
|  | Unknown | 0 | 1 |  |
| **CPSS-Mol score – N(%)** | High | 16 (62%) | 10 (53%) | 0.7 |
|  | Intermediate 1 | 2 (7.7%) | 4 (21%) |  |
|  | Intermediate 2 | 8 (31%) | 3 (16%) |  |
|  | Low | 0 (0%) | 2 (11%) |  |
|  | Unknown | 1 | 1 |  |
| **CMML-stage - N (%)** | CMML-0 | 7 (26%) | 6 (30%) | 0.8 |
|  | CMML-1 | 10 (37%) | 10 (50%) |  |
|  | CMML-2 | 10 (37%) | 4 (20%) |  |

**Supplementary Table 4:** Baseline characteristics of the auto-immune cytopenia subgroup as compared to other SIAD

| **Participants** | | **All other SIAD, N = 35** | **Inflammatory skin disease, N = 12** | **p** |
| --- | --- | --- | --- | --- |
| **Demographic characteristics** | Male sex - N (%) | 17 (49%) | 7 (58%) | >0.9 |
|  | Median age at diagnosis (range) - yrs | 73 (53, 86) | 78 (59, 90) | >0.9 |
| **Laboratory findings** | Leukocytes (IQR) - x 10^9^/L | 13 (9, 21) | 11 (9, 20) | >0.9 |
|  | Neutrophils (IQR) - x 10^9^/L | 7 (4, 11) | 7 (5, 12) | >0.9 |
|  | Platelets (IQR) - x 10^9^/L | 94 (50, 150) | 122 (64, 156) | >0.9 |
|  | Hemoglobin (IQR) - g/dL | 11.70 (10.05, 12.85) | 12.10 (10.55, 13.20) | >0.9 |
|  | Monocytes (IQR) - x 10^9^/L | 2.7 (1.9, 4.5) | 2.1 (1.4, 3.5) | >0.9 |
| **IPSS score - N (%)** | 1 | 9 (31%) | 6 (55%) | >0.9 |
|  | 2 | 12 (41%) | 4 (36%) |  |
|  | 3 | 8 (28%) | 1 (9.1%) |  |
|  | Unknown | 6 | 1 |  |
| **R-IPSS score - N (%)** | 1 | 5 (18%) | 2 (18%) | >0.9 |
|  | 2 | 10 (36%) | 5 (45%) |  |
|  | 3 | 9 (32%) | 2 (18%) |  |
|  | 4 | 4 (14%) | 2 (18%) |  |
|  | Unknown | 7 | 1 |  |
| **Karyotype - N (%)** | Normal | 25 (81%) | 7 (64%) | 0.9 |
|  | 1 other abnormality | 2 (6.5%) | 0 (0%) |  |
|  | 8 trisomy | 2 (6.5%) | 1 (9.1%) |  |
|  | del(20q) | 0 (0%) | 3 (27%) |  |
|  | Complex | 1 (3.2%) | 0 (0%) |  |
|  | Abnormality of chromosome 3 | 1 (3.2%) | 0 (0%) |  |
|  | Unknown | 4 | 1 |  |
| **SIAD - N (%)** | Inflammatory arthritis | 6 (17%) | 3 (25%) | >0.9 |
|  | Pericarditis | 4 (11%) | 2 (17%) | >0.9 |
|  | Autoimmune cytopenia | 19 (54%) | 1 (8.3%) | 0.13 |
|  | Other | 15 (43%) | 2 (17%) | >0.9 |
| **MD vs MP - N (%)** | MD-CMML | 17 (49%) | 7 (58%) | >0.9 |
|  | MP-CMML | 18 (51%) | 5 (42%) |  |
| **CPSS score - N (%)** | High | 4 (12%) | 3 (25%) | >0.9 |
|  | Intermediate 1 | 11 (32%) | 2 (17%) |  |
|  | Intermediate 2 | 12 (35%) | 3 (25%) |  |
|  | Low | 7 (21%) | 4 (33%) |  |
|  | Unknown | 1 | 0 |  |
| **CPSS-Mol score - N (%)** | High | 19 (56%) | 7 (64%) | >0.9 |
|  | Intermediate 1 | 6 (18%) | 0 (0%) |  |
|  | Intermediate 2 | 7 (21%) | 4 (36%) |  |
|  | Low | 2 (5.9%) | 0 (0%) |  |
|  | Unknown | 1 | 1 |  |
| **CMML-OMS - N (%)** | CMML-0 | 9 (26%) | 4 (33%) | >0.9 |
|  | CMML-1 | 14 (40%) | 6 (50%) |  |
|  | CMML-2 | 12 (34%) | 2 (17%) |  |

**Supplementary Table 5:** Baseline characteristics of the inflammatory skin disease subgroup as compared to other SIAD

| **Participants** | | **All other SIAD, N = 38** | **Inflammatory arthritis, N = 9** | **p** |
| --- | --- | --- | --- | --- |
| **Demographic characteristics** | Male sex - N (%) | 19 (50%) | 5 (56%) | >0.9 |
|  | Median age at diagnosis (range) - yrs | 73 (59, 90) | 77 (53, 84) | >0.9 |
| **Laboratory findings** | Leukocytes (IQR) - x 10^9^/L | 13 (9, 22) | 9 (8, 17) | >0.9 |
|  | Neutrophils (IQR) - x 10^9^/L | 7 (5, 12) | 5 (3, 10) | >0.9 |
|  | Platelets (IQR) - x 10^9^/L | 100 (59, 154) | 69 (43, 120) | >0.9 |
|  | Hemoglobin (IQR) - g/dL | 11.75 (10.38, 12.75) | 12.50 (9.70, 13.90) | >0.9 |
|  | Monocytes (IQR) - x 10^9^/L | 2.6 (1.7, 4.5) | 2.5 (1.8, 4.0) | >0.9 |
| **IPSS score - N (%)** | 1 | 13 (41%) | 2 (25%) | >0.9 |
|  | 2 | 11 (34%) | 5 (62%) |  |
|  | 3 | 8 (25%) | 1 (12%) |  |
|  | Unknown | 6 | 1 |  |
| **R-IPSS score - N (%)** | 1 | 6 (19%) | 1 (12%) | >0.9 |
|  | 2 | 11 (35%) | 4 (50%) |  |
|  | 3 | 10 (32%) | 1 (12%) |  |
|  | 4 | 4 (13%) | 2 (25%) |  |
|  | Unknown | 7 | 1 |  |
| **Karyotype - N (%)** | Normal | 28 (82%) | 4 (50%) | 0.9 |
|  | 1 other abnormality | 1 (2.9%) | 1 (12%) |  |
|  | 8 trisomy | 1 (2.9%) | 2 (25%) |  |
|  | del(20q) | 2 (5.9%) | 1 (12%) |  |
|  | Complex | 1 (2.9%) | 0 (0%) |  |
|  | Abnormality of chromosome 3 | 1 (2.9%) | 0 (0%) |  |
|  | Unknown | 4 | 1 |  |
| **SIAD - N (%)** | Inflammatory skin disease | 9 (24%) | 3 (33%) | >0.9 |
|  | Pericarditis | 5 (13%) | 1 (11%) | >0.9 |
|  | Autoimmune cytopenia | 19 (50%) | 1 (11%) | 0.7 |
|  | Other | 14 (37%) | 3 (33%) | >0.9 |
| **MD vs MP - N (%)** | MD-CMML | 19 (50%) | 5 (56%) | >0.9 |
|  | MP-CMML | 19 (50%) | 4 (44%) |  |
| **CPSS score - N (%)** | High | 5 (14%) | 2 (22%) | >0.9 |
|  | Intermediate 1 | 11 (30%) | 2 (22%) |  |
|  | Intermediate 2 | 12 (32%) | 3 (33%) |  |
|  | Low | 9 (24%) | 2 (22%) |  |
|  | Unknown | 1 | 0 |  |
| **CPSS-Mol score - N (%)** | High | 20 (56%) | 6 (67%) | >0.9 |
|  | Intermediate 1 | 5 (14%) | 1 (11%) |  |
|  | Intermediate 2 | 9 (25%) | 2 (22%) |  |
|  | Low | 2 (5.6%) | 0 (0%) |  |
|  | Unknown | 2 | 0 |  |
| **CMML-OMS - N (%)** | CMML-0 | 10 (26%) | 3 (33%) | >0.9 |
|  | CMML-1 | 16 (42%) | 4 (44%) |  |
|  | CMML-2 | 12 (32%) | 2 (22%) |  |

**Supplementary Table 6:** Baseline characteristics of the inflammatory arthritis subgroup as compared to other SIAD.

| **Participants** | | **All other SIAD, N = 41** | **Pericarditis, N = 6** | **p** |
| --- | --- | --- | --- | --- |
| **Demographic characteristics** | Male sex - N (%) | 20 (49%) | 4 (67%) | >0.9 |
|  | Median age at diagnosis (range) - yrs | 74 (57, 90) | 70 (53, 78) | 0.8 |
| **Laboratory findings** | Leukocytes (IQR) - x 10^9^/L | 13 (9, 20) | 14 (11, 50) | >0.9 |
|  | Neutrophils (IQR) - x 10^9^/L | 7 (5, 10) | 7 (5, 20) | >0.9 |
|  | Platelets (IQR) - x 10^9^/L | 96 (50, 158) | 121 (77, 148) | >0.9 |
|  | Hemoglobin (IQR) - g/dL | 12.20 (10.30, 13.20) | 10.20 (9.72, 10.83) | 0.6 |
|  | Monocytes (IQR) - x 10^9^/L | 2.4 (1.5, 3.9) | 4.2 (3.0, 10.8) | 0.6 |
| **IPSS score - N (%)** | 1 | 15 (43%) | 0 (0%) | >0.9 |
|  | 2 | 13 (37%) | 3 (60%) |  |
|  | 3 | 7 (20%) | 2 (40%) |  |
|  | Unknown | 6 | 1 |  |
| **R-IPSS score - N (%)** | 1 | 7 (21%) | 0 (0%) | >0.9 |
|  | 2 | 13 (38%) | 2 (40%) |  |
|  | 3 | 8 (24%) | 3 (60%) |  |
|  | 4 | 6 (18%) | 0 (0%) |  |
|  | Unknown | 7 | 1 |  |
| **Karyotype - N (%)** | Normal | 27 (75%) | 5 (83%) | >0.9 |
|  | 1 other abnormality | 2 (5.6%) | 0 (0%) |  |
|  | 8 trisomy | 3 (8.3%) | 0 (0%) |  |
|  | del(20q) | 2 (5.6%) | 1 (17%) |  |
|  | Complex | 1 (2.8%) | 0 (0%) |  |
|  | Abnormality of chromosome 3 | 1 (2.8%) | 0 (0%) |  |
|  | Unknown | 5 | 0 |  |
| **SIAD - N (%)** | Inflammatory arthritis | 8 (20%) | 1 (17%) | >0.9 |
|  | Inflammatory skin disease | 10 (24%) | 2 (33%) | >0.9 |
|  | Autoimmune cytopenia | 20 (49%) | 0 (0%) | 0.5 |
|  | Other | 15 (37%) | 2 (33%) | >0.9 |
| **MD vs MP - N (%)** | CMML-MD | 22 (54%) | 2 (33%) | >0.9 |
|  | CMML-MP | 19 (46%) | 4 (67%) |  |
| **CPSS score - N (%)** | High | 5 (12%) | 2 (33%) | 0.9 |
|  | Intermediate 1 | 13 (32%) | 0 (0%) |  |
|  | Intermediate 2 | 12 (30%) | 3 (50%) |  |
|  | Low | 10 (25%) | 1 (17%) |  |
|  | Unknown | 1 | 0 |  |
| **CPSS-Mol score - N (%)** | High | 21 (54%) | 5 (83%) | >0.9 |
|  | Intermediate 1 | 6 (15%) | 0 (0%) |  |
|  | Intermediate 2 | 10 (26%) | 1 (17%) |  |
|  | Low | 2 (5.1%) | 0 (0%) |  |
|  | Unknown | 2 | 0 |  |
| **CMML-OMS - N (%)** | CMML-0 | 13 (32%) | 0 (0%) | 0.6 |
|  | CMML-1 | 18 (44%) | 2 (33%) |  |
|  | CMML-2 | 10 (24%) | 4 (67%) |  |

**Supplementary Table 7:** Baseline characteristics of the Pericarditis subgroup as compared to other SIAD.

| **Participants** | | **All, N = 131** | **No SIAD, N = 84** | **SIAD, N = 47** | **p** |
| --- | --- | --- | --- | --- | --- |
| **Mutations - N (%)** | ASXL1 | 51 (39%) | 34 (40%) | 17 (36%) | >0.9 |
|  | BCOR | 7 (5.3%) | 5 (6.0%) | 2 (4.3%) | >0.9 |
|  | BCORL1 | 3 (2.3%) | 2 (2.4%) | 1 (2.1%) | >0.9 |
|  | CALR | 1 (0.8%) | 1 (1.2%) | 0 (0%) | >0.9 |
|  | CBL | 23 (18%) | 12 (14%) | 11 (23%) | 0.8 |
|  | CEBPA | 3 (2.3%) | 2 (2.4%) | 1 (2.1%) | >0.9 |
|  | CSF3R | 1 (0.8%) | 1 (1.2%) | 0 (0%) | >0.9 |
|  | CUX1 | 11 (8.4%) | 4 (4.8%) | 7 (15%) | 0.4 |
|  | DNMT3A | 15 (11%) | 11 (13%) | 4 (8.5%) | >0.9 |
|  | ETNK1 | 3 (2.3%) | 1 (1.2%) | 2 (4.3%) | >0.9 |
|  | EZH2 | 10 (7.6%) | 8 (9.5%) | 2 (4.3%) | >0.9 |
|  | FLT3 | 5 (3.8%) | 3 (3.6%) | 2 (4.3%) | >0.9 |
|  | GATA2 | 1 (0.8%) | 1 (1.2%) | 0 (0%) | >0.9 |
|  | IDH1 | 3 (2.3%) | 0 (0%) | 3 (6.4%) | 0.3 |
|  | IDH2 | 3 (2.3%) | 3 (3.6%) | 0 (0%) | >0.9 |
|  | JAK2 | 10 (7.6%) | 7 (8.3%) | 3 (6.4%) | >0.9 |
|  | KIT | 2 (1.5%) | 1 (1.2%) | 1 (2.1%) | >0.9 |
|  | KRAS | 24 (18%) | 13 (15%) | 11 (23%) | >0.9 |
|  | MPL | 3 (2.3%) | 2 (2.4%) | 1 (2.1%) | >0.9 |
|  | NPM1 | 5 (3.8%) | 3 (3.6%) | 2 (4.3%) | >0.9 |
|  | NRAS | 29 (22%) | 17 (20%) | 12 (26%) | >0.9 |
|  | PHF6 | 6 (4.6%) | 5 (6.0%) | 1 (2.1%) | >0.9 |
|  | PTPN11 | 5 (3.8%) | 3 (3.6%) | 2 (4.3%) | >0.9 |
|  | RUNX1 | 35 (27%) | 21 (25%) | 14 (30%) | >0.9 |
|  | SETBP1 | 7 (5.3%) | 4 (4.8%) | 3 (6.4%) | >0.9 |
|  | SF3B1 | 4 (3.1%) | 2 (2.4%) | 2 (4.3%) | >0.9 |
|  | SH2B3 | 6 (4.6%) | 4 (4.8%) | 2 (4.3%) | >0.9 |
|  | SMC1A | 3 (2.3%) | 2 (2.4%) | 1 (2.1%) | >0.9 |
|  | SRSF2 | 61 (47%) | 38 (45%) | 23 (49%) | >0.9 |
|  | STAG2 | 5 (3.8%) | 4 (4.8%) | 1 (2.1%) | >0.9 |
|  | TET2 | 95 (73%) | 61 (73%) | 34 (72%) | >0.9 |
|  | TP53 | 9 (6.9%) | 7 (8.3%) | 2 (4.3%) | >0.9 |
|  | U2AF1 | 6 (4.6%) | 4 (4.8%) | 2 (4.3%) | >0.9 |
|  | ZRSR2 | 8 (6.1%) | 4 (4.8%) | 4 (8.5%) | >0.9 |
|  |  |  |  |  |  |

**Supplementary Table 8**: Mutational landscape of the entire CMML cohort and according to SIAD status

| **Participants** | | **All other SIAD, N = 27** | **SIAD AI cytopenia , N = 20** | **p** |
| --- | --- | --- | --- | --- |
| **Mutations - N (%)** | ASXL1 | 12 (44%) | 5 (25%) | 0.7 |
|  | BCOR | 1 (3.7%) | 1 (5.0%) | >0.9 |
|  | BCORL1 | 0 (0%) | 1 (5.0%) | 0.8 |
|  | CBL | 6 (22%) | 5 (25%) | >0.9 |
|  | CEBPA | 0 (0%) | 1 (5.0%) | 0.8 |
|  | CUX1 | 4 (15%) | 3 (15%) | >0.9 |
|  | DNMT3A | 2 (7.4%) | 2 (10%) | >0.9 |
|  | ETNK1 | 1 (3.7%) | 1 (5.0%) | >0.9 |
|  | EZH2 | 2 (7.4%) | 0 (0%) | 0.8 |
|  | FLT3 | 1 (3.7%) | 1 (5.0%) | >0.9 |
|  | IDH1 | 2 (7.4%) | 1 (5.0%) | >0.9 |
|  | JAK2 | 1 (3.7%) | 2 (10%) | 0.8 |
|  | KIT | 1 (3.7%) | 0 (0%) | >0.9 |
|  | KRAS | 8 (30%) | 3 (15%) | 0.8 |
|  | MPL | 0 (0%) | 1 (5.0%) | 0.8 |
|  | NPM1 | 2 (7.4%) | 0 (0%) | 0.8 |
|  | NRAS | 9 (33%) | 3 (15%) | 0.7 |
|  | PHF6 | 0 (0%) | 1 (5.0%) | 0.8 |
|  | PTPN11 | 2 (7.4%) | 0 (0%) | 0.8 |
|  | RUNX1 | 9 (33%) | 5 (25%) | 0.8 |
|  | SETBP1 | 3 (11%) | 0 (0%) | 0.8 |
|  | SF3B1 | 1 (3.7%) | 1 (5.0%) | >0.9 |
|  | SH2B3 | 1 (3.7%) | 1 (5.0%) | >0.9 |
|  | SMC1A | 1 (3.7%) | 0 (0%) | >0.9 |
|  | SRSF2 | 13 (48%) | 10 (50%) | >0.9 |
|  | STAG2 | 0 (0%) | 1 (5.0%) | 0.8 |
|  | TET2 | 17 (63%) | 17 (85%) | 0.7 |
|  | TP53 | 0 (0%) | 2 (10%) | 0.7 |
|  | U2AF1 | 1 (3.7%) | 1 (5.0%) | >0.9 |
|  | ZRSR2 | 1 (3.7%) | 3 (15%) | 0.8 |

**Supplementary Table 9:** Mutational status of autoimmune cytopenia versus the other SIAD

| **Participants** | | **All other SIAD, N = 41** | **Pericarditis**  **N = 6** | **p** |
| --- | --- | --- | --- | --- |
| **Mutations - N (%)** | ASXL1 | 14 (34%) | 3 (50%) | >0.9 |
|  | BCOR | 2 (4.9%) | 0 (0%) | >0.9 |
|  | BCORL1 | 1 (2.4%) | 0 (0%) | >0.9 |
|  | CBL | 9 (22%) | 2 (33%) | >0.9 |
|  | CEBPA | 1 (2.4%) | 0 (0%) | >0.9 |
|  | CUX1 | 6 (15%) | 1 (17%) | >0.9 |
|  | DNMT3A | 4 (9.8%) | 0 (0%) | >0.9 |
|  | ETNK1 | 2 (4.9%) | 0 (0%) | >0.9 |
|  | EZH2 | 2 (4.9%) | 0 (0%) | >0.9 |
|  | FLT3 | 2 (4.9%) | 0 (0%) | >0.9 |
|  | IDH1 | 2 (4.9%) | 1 (17%) | >0.9 |
|  | JAK2 | 3 (7.3%) | 0 (0%) | >0.9 |
|  | KIT | 1 (2.4%) | 0 (0%) | >0.9 |
|  | KRAS | 7 (17%) | 4 (67%) | 0.5 |
|  | MPL | 1 (2.4%) | 0 (0%) | >0.9 |
|  | NPM1 | 2 (4.9%) | 0 (0%) | >0.9 |
|  | NRAS | 9 (22%) | 3 (50%) | 0.9 |
|  | PHF6 | 1 (2.4%) | 0 (0%) | >0.9 |
|  | PTPN11 | 2 (4.9%) | 0 (0%) | >0.9 |
|  | RUNX1 | 13 (32%) | 1 (17%) | >0.9 |
|  | SETBP1 | 2 (4.9%) | 1 (17%) | >0.9 |
|  | SF3B1 | 2 (4.9%) | 0 (0%) | >0.9 |
|  | SH2B3 | 2 (4.9%) | 0 (0%) | >0.9 |
|  | SMC1A | 0 (0%) | 1 (17%) | 0.8 |
|  | SRSF2 | 19 (46%) | 4 (67%) | >0.9 |
|  | STAG2 | 1 (2.4%) | 0 (0%) | >0.9 |
|  | TET2 | 30 (73%) | 4 (67%) | >0.9 |
|  | TP53 | 2 (4.9%) | 0 (0%) | >0.9 |
|  | U2AF1 | 2 (4.9%) | 0 (0%) | >0.9 |
|  | ZRSR2 | 4 (9.8%) | 0 (0%) | >0.9 |

**Supplementary Table 10:** Mutational status of pericarditis versus the other SIAD

| **Participants** | | **All other SIAD, N = 35** | **Inflammatory skin disease**  **N = 12** | **p** |
| --- | --- | --- | --- | --- |
| **Mutations - N (%)** | ASXL1 | 11 (31%) | 6 (50%) | >0.9 |
|  | BCOR | 1 (2.9%) | 1 (8.3%) | >0.9 |
|  | BCORL1 | 1 (2.9%) | 0 (0%) | >0.9 |
|  | CBL | 7 (20%) | 4 (33%) | >0.9 |
|  | CEBPA | 1 (2.9%) | 0 (0%) | >0.9 |
|  | CUX1 | 3 (8.6%) | 4 (33%) | 0.9 |
|  | DNMT3A | 4 (11%) | 0 (0%) | >0.9 |
|  | ETNK1 | 2 (5.7%) | 0 (0%) | >0.9 |
|  | EZH2 | 1 (2.9%) | 1 (8.3%) | >0.9 |
|  | FLT3 | 2 (5.7%) | 0 (0%) | >0.9 |
|  | IDH1 | 3 (8.6%) | 0 (0%) | >0.9 |
|  | JAK2 | 2 (5.7%) | 1 (8.3%) | >0.9 |
|  | KIT | 0 (0%) | 1 (8.3%) | >0.9 |
|  | KRAS | 9 (26%) | 2 (17%) | >0.9 |
|  | MPL | 0 (0%) | 1 (8.3%) | >0.9 |
|  | NPM1 | 2 (5.7%) | 0 (0%) | >0.9 |
|  | NRAS | 7 (20%) | 5 (42%) | >0.9 |
|  | PHF6 | 1 (2.9%) | 0 (0%) | >0.9 |
|  | PTPN11 | 1 (2.9%) | 1 (8.3%) | >0.9 |
|  | RUNX1 | 9 (26%) | 5 (42%) | >0.9 |
|  | SETBP1 | 1 (2.9%) | 2 (17%) | >0.9 |
|  | SF3B1 | 2 (5.7%) | 0 (0%) | >0.9 |
|  | SH2B3 | 1 (2.9%) | 1 (8.3%) | >0.9 |
|  | SMC1A | 1 (2.9%) | 0 (0%) | >0.9 |
|  | SRSF2 | 16 (46%) | 7 (58%) | >0.9 |
|  | STAG2 | 1 (2.9%) | 0 (0%) | >0.9 |
|  | TET2 | 26 (74%) | 8 (67%) | >0.9 |
|  | TP53 | 1 (2.9%) | 1 (8.3%) | >0.9 |
|  | U2AF1 | 1 (2.9%) | 1 (8.3%) | >0.9 |
|  | ZRSR2 | 4 (11%) | 0 (0%) | >0.9 |

**Supplementary Table 11:** Mutational status of inflammatory skin disease versus the other SIAD

| **Participants** | | **All other SIAD, N = 38** | **Inflammatory arthritis, N = 9** | **p** |
| --- | --- | --- | --- | --- |
| **Mutations - N (%)** | ASXL1 | 13 (34%) | 4 (44%) | >0.9 |
|  | BCOR | 2 (5.3%) | 0 (0%) | >0.9 |
|  | BCORL1 | 1 (2.6%) | 0 (0%) | >0.9 |
|  | CBL | 8 (21%) | 3 (33%) | >0.9 |
|  | CEBPA | 1 (2.6%) | 0 (0%) | >0.9 |
|  | CUX1 | 5 (13%) | 2 (22%) | >0.9 |
|  | DNMT3A | 4 (11%) | 0 (0%) | >0.9 |
|  | ETNK1 | 1 (2.6%) | 1 (11%) | >0.9 |
|  | EZH2 | 1 (2.6%) | 1 (11%) | >0.9 |
|  | FLT3 | 2 (5.3%) | 0 (0%) | >0.9 |
|  | IDH1 | 2 (5.3%) | 1 (11%) | >0.9 |
|  | JAK2 | 1 (2.6%) | 2 (22%) | 0.7 |
|  | KIT | 1 (2.6%) | 0 (0%) | >0.9 |
|  | KRAS | 10 (26%) | 1 (11%) | >0.9 |
|  | MPL | 0 (0%) | 1 (11%) | >0.9 |
|  | NPM1 | 2 (5.3%) | 0 (0%) | >0.9 |
|  | NRAS | 12 (32%) | 0 (0%) | 0.7 |
|  | PHF6 | 1 (2.6%) | 0 (0%) | >0.9 |
|  | PTPN11 | 1 (2.6%) | 1 (11%) | >0.9 |
|  | RUNX1 | 10 (26%) | 4 (44%) | >0.9 |
|  | SETBP1 | 1 (2.6%) | 2 (22%) | 0.7 |
|  | SF3B1 | 2 (5.3%) | 0 (0%) | >0.9 |
|  | SH2B3 | 2 (5.3%) | 0 (0%) | >0.9 |
|  | SMC1A | 1 (2.6%) | 0 (0%) | >0.9 |
|  | SRSF2 | 19 (50%) | 4 (44%) | >0.9 |
|  | STAG2 | 1 (2.6%) | 0 (0%) | >0.9 |
|  | TET2 | 30 (79%) | 4 (44%) | 0.7 |
|  | TP53 | 1 (2.6%) | 1 (11%) | >0.9 |
|  | U2AF1 | 2 (5.3%) | 0 (0%) | >0.9 |
|  | ZRSR2 | 4 (11%) | 0 (0%) | >0.9 |

**Supplementary Table 12:** Mutational status of inflammatory arthritis versus the other SIAD

| **Overall survival whole cohort** | | | | | | | | | | |
| --- | --- | --- | --- | --- | --- | --- | --- | --- | --- | --- |
| Univariate Cox model |  |  |  |  | Multivariate Cox model | |  |  |  |  |
|  | HR | 95% CI | p | Adjusted  p |  | HR | | 95% CI | p |  |
| SIAD | 1.18 | 0.75, 1.83 | 0.5 | 0.77 | MD CMML | 0.46 | | 0.29-0.74 | 0.0012 |  |
| Inflammatory arthritis | 1.30 | 0.56, 3.00 | 0.5 | 0.77 | TET2 | 0.43 | | 0.25-0.74 | 0.002 |  |
| Inflammatory skin disease | 1.16 | 0.58, 2.32 | 0.7 | 0.82 | RUNX1 | 1.67 | | 0.95-2.93 | 0.072 |  |
| Pericarditis | 1.58 | 0.57, 4.35 | 0.4 | 0.73 | SRSF2 | 1.65 | | 0.96-2.83 | 0.070 |  |
| Autoimmune cytopenia | 0.63 | 0.33, 1.20 | 0.2 | 0.45 |  |  | |  |  |  |
| MD CMML | 0.44 | 0.28, 0.68 | <0.001 | 0.0099 |  |  | |  |  |  |
| CPSS Int-1 | 0.49 | 0.26, 0.92 | 0.025 | 0.19 |  |  | |  |  |  |
| CPSS Int-2 | 0.51 | 0.28, 0.93 | 0.029 | 0.19 |  |  | |  |  |  |
| CPSS Low | 0.41 | 0.21, 0.82 | 0.011 | 0.15 |  |  | |  |  |  |
| CPSS mol Int-1 | 0.54 | 0.29, 0.99 | 0.047 | 0.21 |  |  | |  |  |  |
| CPSS mol Int-2 | 0.64 | 0.36, 1.15 | 0.14 | 0.41 |  |  | |  |  |  |
| CPSS mol Low | 0.18 | 0.04, 0.73 | 0.016 | 0.15 |  |  | |  |  |  |
| ASXL1 | 1.58 | 1.02, 2.45 | 0.042 | 0.21 |  |  | |  |  |  |
| BCOR | 1.80 | 0.78, 4.17 | 0.2 | 0.46 |  |  | |  |  |  |
| BCORL1 | 0.82 | 0.26, 2.63 | 0.7 | 0.85 |  |  | |  |  |  |
| CALR | 0.67 | 0.09, 4.86 | 0.7 | 0.82 |  |  | |  |  |  |
| CBL | 1.57 | 0.92, 2.68 | 0.10 | 0.34 |  |  | |  |  |  |
| CEBPA | 1.85 | 0.45, 7.59 | 0.4 | 0.73 |  |  | |  |  |  |
| CSF3R | 0.00 | 0.00, Inf | >0.9 | 1.00 |  |  | |  |  |  |
| CUX1 | 1.32 | 0.63, 2.75 | 0.5 | 0.77 |  |  | |  |  |  |
| DNMT3A | 1.32 | 0.71, 2.46 | 0.4 | 0.73 |  |  | |  |  |  |
| ETNK1 | 0.66 | 0.20, 2.20 | 0.5 | 0.77 |  |  | |  |  |  |
| EZH2 | 1.40 | 0.67, 2.90 | 0.4 | 0.73 |  |  | |  |  |  |
| FLT3 | 2.46 | 0.99, 6.12 | 0.052 | 0.21 |  |  | |  |  |  |
| GATA2 | 0.79 | 0.11, 5.75 | 0.8 | 0.90 |  |  | |  |  |  |
| IDH1 | 3.20 | 0.99, 10.4 | 0.052 | 0.21 |  |  | |  |  |  |
| IDH2 | 0.50 | 0.07, 3.61 | 0.5 | 0.77 |  |  | |  |  |  |
| JAK2 | 1.30 | 0.63, 2.72 | 0.5 | 0.77 |  |  | |  |  |  |
| KIT | 1.65 | 0.40, 6.76 | 0.5 | 0.77 |  |  | |  |  |  |
| KRAS | 1.08 | 0.61, 1.92 | 0.8 | 0.90 |  |  | |  |  |  |
| MPL | 1.36 | 0.33, 5.56 | 0.7 | 0.82 |  |  | |  |  |  |
| No_mut | 0.63 | 0.09, 4.56 | 0.6 | 0.82 |  |  | |  |  |  |
| NPM1 | 1.95 | 0.71, 5.36 | 0.2 | 0.50 |  |  | |  |  |  |
| NRAS | 1.27 | 0.76, 2.11 | 0.4 | 0.73 |  |  | |  |  |  |
| PHF6 | 1.27 | 0.55, 2.93 | 0.6 | 0.77 |  |  | |  |  |  |
| PTPN11 | 0.99 | 0.24, 4.03 | >0.9 | 1.00 |  |  | |  |  |  |
| RUNX1 | 1.76 | 1.11, 2.79 | 0.017 | 0.15 |  |  | |  |  |  |
| SETBP1 | 1.77 | 0.77, 4.09 | 0.18 | 0.44 |  |  | |  |  |  |
| SF3B1 | 1.43 | 0.45, 4.56 | 0.5 | 0.77 |  |  | |  |  |  |
| SH2B3 | 0.97 | 0.42, 2.25 | >0.9 | 0.99 |  |  | |  |  |  |
| SMC1A | 0.87 | 0.21, 3.60 | 0.9 | 0.91 |  |  | |  |  |  |
| SRSF2 | 1.89 | 1.22, 2.92 | 0.004 | 0.09 |  |  | |  |  |  |
| STAG2 | 2.30 | 0.84, 6.34 | 0.11 | 0.34 |  |  | |  |  |  |
| TET2 | 0.66 | 0.41, 1.08 | 0.10 | 0.34 |  |  | |  |  |  |
| TP53 | 1.48 | 0.68, 3.23 | 0.3 | 0.73 |  |  | |  |  |  |
| U2AF1 | 1.25 | 0.46, 3.44 | 0.7 | 0.82 |  |  | |  |  |  |
| ZRSR2 | 1.33 | 0.53, 3.30 | 0.5 | 0.77 |  |  | |  |  |  |

**Supplementary Table 13:** Univariate and multivariate Cox model analysis of the role of clinical, biological, and mutational on OS of the whole cohort.

| **Progression free survival whole cohort** | | | | | | | | |
| --- | --- | --- | --- | --- | --- | --- | --- | --- |
| Univariate Cox model |  |  |  |  | Multivariate Cox model | |  |  |
|  | HR | 95% CI | p | Adjusted  p |  | HR | 95% CI | p |
| SIAD | 1.13 | 0.73, 1.73 | 0.6 | 0.75 | MD CMML | 0.53 | 0.34-0.83 | 0.0052 |
| Inflammatory arthritis | 1.30 | 0.60, 2.82 | 0.5 | 0.72 | RUNX1 | 2.23 | 1.37-3.64 | 0.0013 |
| Inflammatory skin disease | 1.19 | 0.62, 2.31 | 0.6 | 0.75 | ASXL1 | 1.54 | 0.99-2.41 | 0.057 |
| Pericarditis | 1.51 | 0.55, 4.15 | 0.4 | 0.71 | DNMT3A | 1.77 | 0.93- 3.37 | 0.082 |
| Autoimmune cytopenia | 0.64 | 0.34, 1.18 | 0.15 | 0.33 |  |  |  |  |
| MD-CMML | 0.48 | 0.31, 0.73 | <0.001 | 0.006 |  |  |  |  |
| CPSS Int-1 | 0.36 | 0.20, 0.65 | <0.001 | 0.006 |  |  |  |  |
| CPSS Int-2 | 0.43 | 0.24, 0.77 | 0.004 | 0.02 |  |  |  |  |
| CPSS Low | 0.29 | 0.15, 0.56 | <0.001 | 0.004 |  |  |  |  |
| CPSS mol Int-1 | 0.38 | 0.21, 0.71 | 0.002 | 0.013 |  |  |  |  |
| CPSS mol Int-2 | 0.64 | 0.38, 1.10 | 0.11 | 0.26 |  |  |  |  |
| CPSS mol Low | 0.13 | 0.03, 0.53 | 0.004 | 0.02 |  |  |  |  |
| ASXL1 | 1.87 | 1.23, 2.85 | 0.004 | 0.018 |  |  |  |  |
| BCOR | 2.06 | 0.94, 4.50 | 0.070 | 0.21 |  |  |  |  |
| BCORL1 | 0.99 | 0.31, 3.19 | >0.9 | 0.99 |  |  |  |  |
| CALR | 0.57 | 0.08, 4.11 | 0.6 | 0.75 |  |  |  |  |
| CBL | 1.41 | 0.84, 2.38 | 0.2 | 0.38 |  |  |  |  |
| CEBPA | 22.2 | 5.67, 87.1 | <0.001 | 0.00039 |  |  |  |  |
| CSF3R | 16.3 | 2.03, 131 | 0.009 | 0.032 |  |  |  |  |
| CUX1 | 1.27 | 0.61, 2.63 | 0.5 | 0.72 |  |  |  |  |
| DNMT3A | 1.66 | 0.91, 3.01 | 0.10 | 0.25 |  |  |  |  |
| ETNK1 | 0.59 | 0.18, 1.96 | 0.4 | 0.68 |  |  |  |  |
| EZH2 | 1.64 | 0.82, 3.27 | 0.2 | 0.34 |  |  |  |  |
| FLT3 | 3.92 | 1.57, 9.78 | 0.003 | 0.018 |  |  |  |  |
| GATA2 | 2.01 | 0.28, 14.6 | 0.5 | 0.72 |  |  |  |  |
| IDH1 | 2.54 | 0.79, 8.16 | 0.12 | 0.26 |  |  |  |  |
| IDH2 | 0.39 | 0.05, 2.84 | 0.4 | 0.64 |  |  |  |  |
| JAK2 | 1.20 | 0.58, 2.49 | 0.62 | 0.76 |  |  |  |  |
| KIT | 1.21 | 0.30, 4.95 | 0.8 | 0.91 |  |  |  |  |
| KRAS | 1.06 | 0.60, 1.84 | 0.8 | 0.94 |  |  |  |  |
| MPL | 1.06 | 0.26, 4.34 | >0.9 | 0.98 |  |  |  |  |
| No_mut | 0.50 | 0.07, 3.58 | 0.5 | 0.72 |  |  |  |  |
| NPM1 | 5.92 | 2.32, 15.1 | <0.001 | 0.0038 |  |  |  |  |
| NRAS | 1.53 | 0.95, 2.47 | 0.079 | 0.22 |  |  |  |  |
| PHF6 | 1.04 | 0.45, 2.38 | >0.9 | 0.98 |  |  |  |  |
| PTPN11 | 6.15 | 2.16, 17.5 | <0.001 | 0.0060 |  |  |  |  |
| RUNX1 | 1.79 | 1.15, 2.79 | 0.010 | 0.036 |  |  |  |  |
| SETBP1 | 3 | 1.35, 6.69 | 0.0070 | 0.027 |  |  |  |  |
| SF3B1 | 1.11 | 0.35, 3.53 | 0.9 | 0.94 |  |  |  |  |
| SH2B3 | 0.82 | 0.35, 1.89 | 0.64 | 0.77 |  |  |  |  |
| SMC1A | 1.48 | 0.46, 4.77 | 0.52 | 0.72 |  |  |  |  |
| SRSF2 | 1.51 | 1.00, 2.29 | 0.052 | 0.17 |  |  |  |  |
| STAG2 | 1.85 | 0.68, 5.08 | 0.23 | 0.43 |  |  |  |  |
| TET2 | 0.68 | 0.43, 1.09 | 0.11 | 0.26 |  |  |  |  |
| TP53 | 1.17 | 0.54, 2.54 | 0.69 | 0.82 |  |  |  |  |
| U2AF1 | 0.97 | 0.35, 2.66 | 0.96 | 0.98 |  |  |  |  |
| ZRSR2 | 1.37 | 0.55, 3.40 | 0.50 | 0.72 |  |  |  |  |

**Supplementary Table 14:** Univariate and multivariate Cox model analysis of the role of clinical, biological, and mutational on PFS of the whole cohort

| **Overall survival SIAD patients** | | | | | | | | |
| --- | --- | --- | --- | --- | --- | --- | --- | --- |
| Univariate Cox model |  |  |  |  | Multivariate Cox model | |  |  |
|  | HR | 95% CI | p | Adjusted  p |  | HR | 95% CI | p |
| Inflammatory arthritis | 1.11 | 0.45, 2.76 | 0.8 | 0.88 | AI Cytopenia | 0.43 | 0.19-0.99 | 0.047 |
| Inflammatory skin disease | 1.01 | 0.46, 2.21 | >0.9 | >0.9 | ASXL1 | 1.81 | 0.87-3.77 | 0.1 |
| Pericarditis | 1.42 | 0.49, 4.14 | 0.5 | 0.84 | DNMT3A | 10.81 | 3.04-38.4 | 0.00023 |
| Autoimmune cytopenia | 0.39 | 0.17, 0.87 | 0.021 | 0.16 |  |  |  |  |
| MD-CMML | 0.70 | 0.34, 1.42 | 0.3 | 0.71 |  |  |  |  |
| CPSS Int-1 | 0.70 | 0.23, 2.12 | 0.5 | 0.84 |  |  |  |  |
| CPSS Int-2 | 0.75 | 0.25, 2.24 | 0.6 | 0.84 |  |  |  |  |
| CPSS Low | 0.53 | 0.16, 1.76 | 0.3 | 0.7 |  |  |  |  |
| CPSS mol Int-1 | 0.29 | 0.07, 1.26 | 0.10 | 0.40 |  |  |  |  |
| CPSS mol Int-2 | 1.13 | 0.49, 2.58 | 0.8 | 0.88 |  |  |  |  |
| CPSS mol Low | 1.48 | 0.34, 6.48 | 0.6 | 0.84 |  |  |  |  |
| ASXL1 | 2.00 | 0.99, 4.04 | 0.052 | 0.35 |  |  |  |  |
| BCOR | 3.52 | 0.80, 15.6 | 0.10 | 0.40 |  |  |  |  |
| BCORL1 | 2.39 | 0.32, 18.1 | 0.4 | 0.80 |  |  |  |  |
| CBL | 1.27 | 0.57, 2.84 | 0.6 | 0.84 |  |  |  |  |
| CUX1 | 1.83 | 0.74, 4.54 | 0.2 | 0.58 |  |  |  |  |
| DNMT3A | 6.65 | 2.08, 21.3 | 0.001 | 0.028 |  |  |  |  |
| ETNK1 | 0.63 | 0.14, 2.81 | 0.5 | 0.84 |  |  |  |  |
| EZH2 | 3.76 | 0.84, 16.8 | 0.082 | 0.40 |  |  |  |  |
| FLT3 | 24.1 | 3.97, 147 | <0.001 | 0.022 |  |  |  |  |
| IDH1 | 3.05 | 0.88, 10.6 | 0.079 | 0.40 |  |  |  |  |
| JAK2 | 0.71 | 0.21, 0.39 | 0.6 | 0.84 |  |  |  |  |
| KIT | 1.47 | 0.20, 10.9 | 0.7 | 0.86 |  |  |  |  |
| KRAS | 0.88 | 0.36, 2.16 | 0.8 | 0.88 |  |  |  |  |
| MPL | 0.82 | 0.11, 6.10 | 0.8 | 0.88 |  |  |  |  |
| NPM1 | 11.3 | 2.05, 62.2 | 0.005 | 0.072 |  |  |  |  |
| NRAS | 1.19 | 0.53, 2.68 | 0.7 | 0.86 |  |  |  |  |
| PHF6 | 4.37 | 0.55, 34.5 | 0.2 | 0.58 |  |  |  |  |
| PTPN11 | 2.19 | 0.29, 16.6 | 0.4 | 0.83 |  |  |  |  |
| RUNX1 | 1.68 | 0.78, 3.62 | 0.2 | 0.58 |  |  |  |  |
| SETBP1 | 1.22 | 0.28, 5.22 | 0.8 | 0.88 |  |  |  |  |
| SF3B1 | 2.29 | 0.53, 9.78 | 0.3 | 0.66 |  |  |  |  |
| SH2B3 | 1.42 | 0.33, 6.10 | 0.6 | 0.85 |  |  |  |  |
| SMC1A | 43.5 | 2.72, 695 | 0.008 | 0.076 |  |  |  |  |
| SRSF2 | 1.58 | 0.76, 3.27 | 0.2 | 0.58 |  |  |  |  |
| STAG2 | 2.39 | 0.32, 18.1 | 0.4 | 0.79 |  |  |  |  |
| TET2 | 0.60 | 0.28, 1.32 | 0.2 | 0.58 |  |  |  |  |
| TP53 | 1.59 | 0.37, 6.79 | 0.5 | 0.84 |  |  |  |  |
| U2AF1 | 1.47 | 0.20, 10.9 | 0.7 | 0.86 |  |  |  |  |
| ZRSR2 | 1.07 | 0.25, 4.54 | >0.9 | >0.9 |  |  |  |  |

**Supplementary Table 15:** Univariate and multivariate Cox model analysis of the role of clinical, biological, and mutational on OS of SIAD patients.

| **Progression free survival SIAD patients** | | | | | | | | | |
| --- | --- | --- | --- | --- | --- | --- | --- | --- | --- |
|  |  |  |  |  |  | | | |  |
| Univariate Cox model |  |  |  |  | Multivariate Cox model | | | |  |
|  | HR | 95% CI | p | Adjusted p |  | HR | 95% CI | p |  |
| Inflammatory arthritis | 1.05 | 0.45, 2.45 | >0.9 | >0.9 | ASXL1 | 2.37 | 1.16-4.84 | 0.018 |  |
| Inflammatory skin disease | 0.85 | 0.39, 1.87 | 0.7 | >0.9 | CUX1 | 2.86 | 1.12-7.31 | 0.029 |  |
| Pericarditis | 1.37 | 0.47, 3.97 | 0.6 | >0.9 | DNMT3A | 6.93 | 2.11-22.8 | 0.0014 |  |
| Autoimmune cytopenia | 0.58 | 0.28, 1.21 | 0.15 | 0.50 |  |  |  | |  |
| MD CMML | 1.05 | 0.52, 2.11 | 0.9 | >0.9 |  |  |  | |  |
| CPSS Int-1 | 0.50 | 0.18, 1.43 | 0.2 | 0.51 |  |  |  | |  |
| CPSS Int-2 | 0.51 | 0.18, 1.43 | 0.2 | 0.51 |  |  |  | |  |
| CPSS Low | 0.29 | 0.09, 0.93 | 0.037 | 0.21 |  |  |  | |  |
| CPSS mol Int-1 | 0.19 | 0.04, 0.84 | 0.028 | 0.19 |  |  |  | |  |
| CPSS mol Int-2 | 0.65 | 0.28, 1.51 | 0.3 | 0.62 |  |  |  | |  |
| CPSS mol Low | 0.88 | 0.20, 3.79 | 0.9 | >0.9 |  |  |  | |  |
| ASXL1 | 1.99 | 0.99, 3.97 | 0.052 | 0.24 |  |  |  | |  |
| BCOR | 2.91 | 0.66, 12.8 | 0.2 | 0.51 |  |  |  | |  |
| BCORL1 | 2.02 | 0.27, 15.3 | 0.5 | 0.85 |  |  |  | |  |
| CBL | 0.85 | 0.38, 1.89 | 0.7 | 0.92 |  |  |  | |  |
| CEBPA | 41.0 | 2.56, 655 | 0.009 | 0.12 |  |  |  | |  |
| CUX1 | 2.52 | 1.01, 6.30 | 0.048 | 0.24 |  |  |  | |  |
| DNMT3A | 5.75 | 1.84, 18.0 | 0.003 | 0.054 |  |  |  | |  |
| ETNK1 | 1.61 | 0.21, 12.0 | 0.6 | >0.9 |  |  |  | |  |
| EZH2 | 2.44 | 0.56, 10.6 | 0.2 | 0.51 |  |  |  | |  |
| FLT3 | 5 356 214 000 029 | 0.00, Inf | >0.9 | >0.9 |  |  |  | |  |
| IDH1 | 2.34 | 0.69, 7.95 | 0.2 | 0.51 |  |  |  | |  |
| JAK2 | 0.80 | 0.24, 0.68 | 0.7 | >0.9 |  |  |  | |  |
| KIT | 0.93 | 0.13, 6.88 | >0.9 | >0.9 |  |  |  | |  |
| KRAS | 1.05 | 0.42, 2.61 | >0.9 | >0.9 |  |  |  | |  |
| MPL | 0.64 | 0.09, 4.80 | 0.7 | >0.9 |  |  |  | |  |
| NPM1 | 33.3 | 4.60, 241 | <0.001 | 0.021 |  |  |  | |  |
| NRAS | 0.82 | 0.37, 1.83 | 0.6 | >0.9 |  |  |  | |  |
| PHF6 | 2.89 | 0.38, 22.2 | 0.3 | 0.62 |  |  |  | |  |
| PTPN11 | 2.99 | 0.39, 23.2 | 0.3 | 0.62 |  |  |  | |  |
| RUNX1 | 1.77 | 0.84, 3.72 | 0.13 | 0.50 |  |  |  | |  |
| SETBP1 | 2.92 | 0.86, 9.98 | 0.087 | 0.36 |  |  |  | |  |
| SF3B1 | 1.72 | 0.40, 7.29 | 0.5 | 0.85 |  |  |  | |  |
| SH2B3 | 1.05 | 0.24, 4.53 | >0.9 | >0.9 |  |  |  | |  |
| SMC1A | 20.2 | 1.83, 222 | 0.014 | 0.13 |  |  |  | |  |
| SRSF2 | 1.53 | 0.75, 3.15 | 0.2 | 0.51 |  |  |  | |  |
| STAG2 | 2.02 | 0.27, 15.3 | 0.5 | 0.85 |  |  |  | |  |
| TET2 | 0.39 | 0.18, 0.84 | 0.016 | 0.13 |  |  |  | |  |
| TP53 | 1.22 | 0.28, 5.22 | 0.8 | >0.9 |  |  |  | |  |
| U2AF1 | 0.93 | 0.13, 6.88 | >0.9 | >0.9 |  |  |  | |  |
| ZRSR2 | 0.77 | 0.18, 3.24 | 0.7 | >0.9 |  |  |  | |  |

**Supplementary Table 16:** Univariate and multivariate Cox model analysis of the role of clinical, biological, and mutational on PFS of SIAD patients.

**Figures**


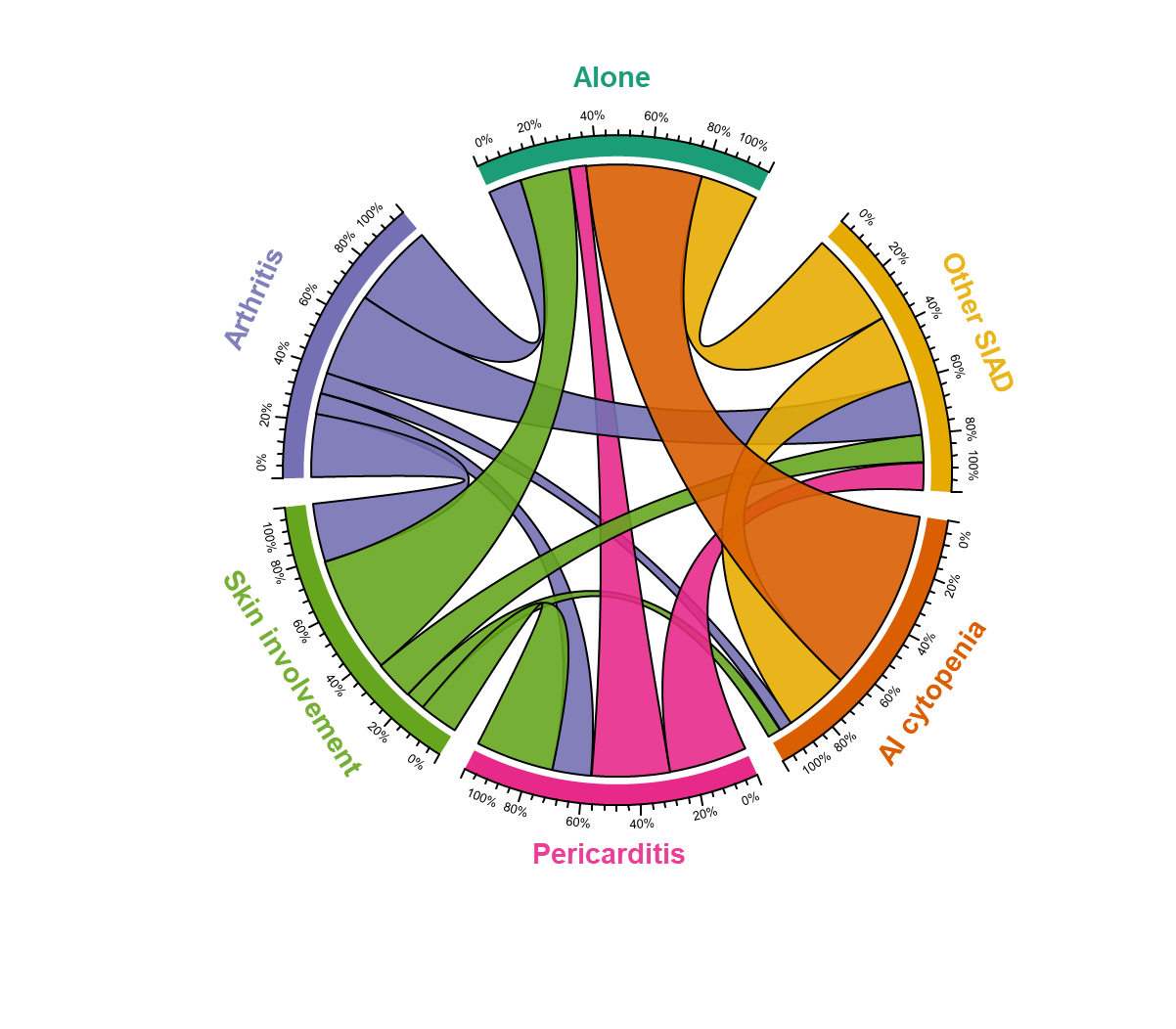


**Supplementary Figure 1**: Circos plot summarizing the associations between the different types of SIAD. The strength of the association is represented as a percentage of the total of each SIAD.


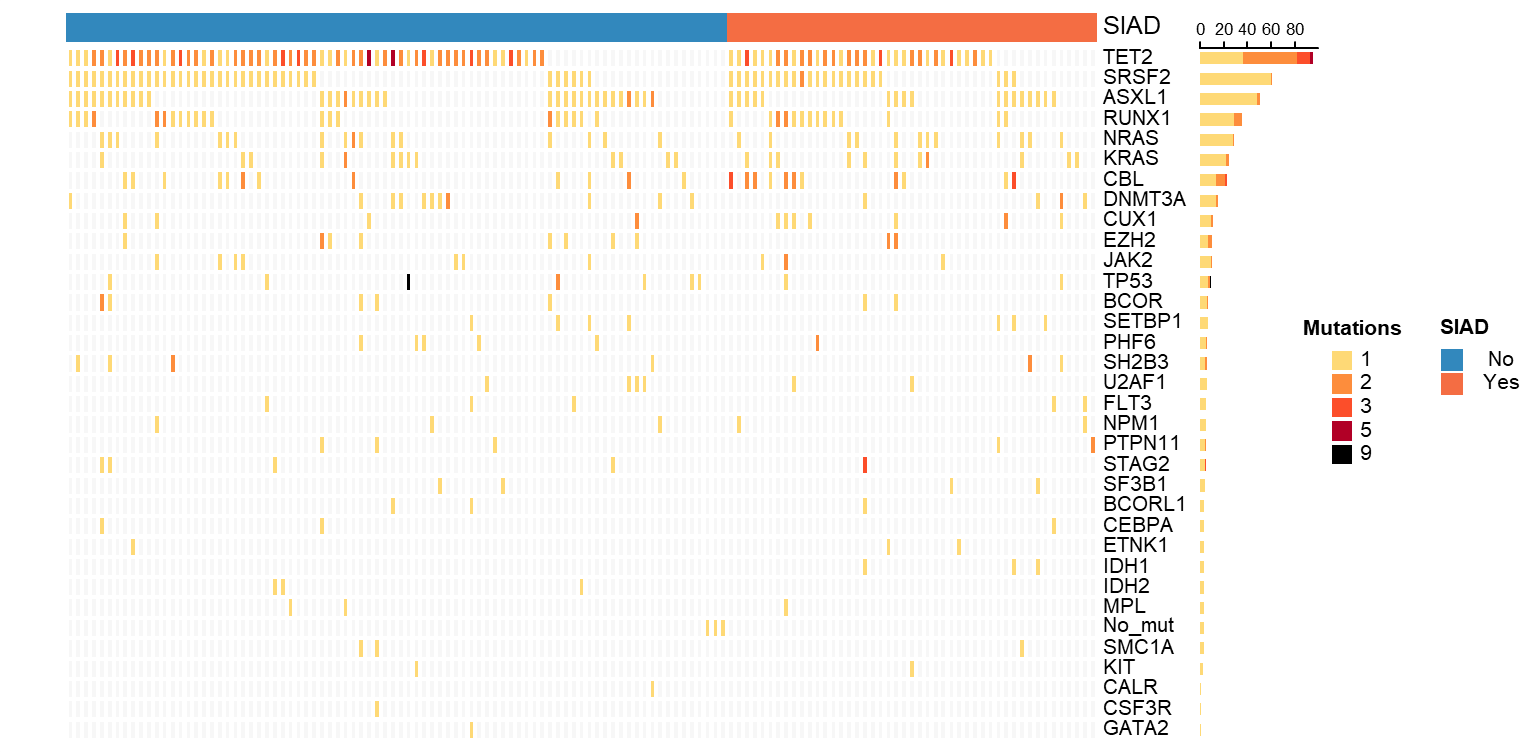


**Supplementary Figure 2:** Oncoplot representing the mutational landscape of the cohort. Mutations are ordered from top to bottom, from the most frequent to the least frequent (right part of the oncoplot). The number of mutations is represented in a gradient from yellow to black

**Supplementary Figure 3:** OS and PFS of the whole cohort according to CPSS and CPSS-Mol scores (Survival curves were compared with the Log-rank test).

Distribution of patients in the different groups of the CPSS risk score: 22 High, 37 intermediate-2, 37 Intermediate-1, and 32 Low.

Distribution of patients in the different groups of the CPSS-Mol risk score: 62 High, 27 intermediate-2, 26 Intermediate-1, and 10 Low.
